# Chk2 and REGγ-dependent DBC1 regulation in DNA damage induced apoptosis

**DOI:** 10.1093/nar/gku1065

**Published:** 2014-10-31

**Authors:** Martina Magni, Vincenzo Ruscica, Giacomo Buscemi, Ja-Eun Kim, Benjamin Tamilselvan Nachimuthu, Enrico Fontanella, Domenico Delia, Laura Zannini

**Affiliations:** 1 Department of Experimental Oncology, Fondazione IRCCS Istituto Nazionale dei Tumori, 20133 Milan, Italy; 2 Department of Biosciences, University of Milan, 20133 Milan, Italy; 3 Department of Pharmacology, School of Medicine, Kyung Hee University, Seoul 130-701, Republic of Korea; 4 Istituto di Genetica Molecolare, Consiglio Nazionale delle Ricerche (IGM-CNR), 27100 Pavia, Italy

## Abstract

Human DBC1 (Deleted in Breast Cancer 1; KIAA1967; CCAR2) is a protein implicated in the regulation of apoptosis, transcription and histone modifications. Upon DNA damage, DBC1 is phosphorylated by ATM/ATR on Thr454 and this modification increases its inhibitory interaction with SIRT1, leading to p53 acetylation and p53-dependent apoptosis. Here, we report that the inhibition of SIRT1 by DBC1 in the DNA damage response (DDR) also depends on Chk2, the transducer kinase that is activated by ATM upon DNA lesions and contributes to the spreading of DNA damage signal. Indeed we found that inactivation of Chk2 reduces DBC1-SIRT1 binding, thus preventing p53 acetylation and DBC1-induced apoptosis. These events are mediated by Chk2 phosphorylation of the 11S proteasome activator REGγ on Ser247, which increases REGγ-DBC1 interaction and SIRT1 inhibition. Overall our results clarify the mechanisms underlying the DBC1-dependent SIRT1 inhibition and link, for the first time, Chk2 and REGγ to the ATM-DBC1-SIRT1 axis.

## INTRODUCTION

In order to preserve genomic stability, eukaryotic cells have evolved a complex network of signaling pathways, collectively known as DNA damage response (DDR), that are activated when cells are exposed to genotoxic lesions (1).

Key components of the DDR cascade in response to double-strand breaks (DSBs) are the apical kinase ATM (Ataxia Telangiectasia Mutated) and its target and effector checkpoint kinase Chk2 (2). Following DNA damage, ATM phosphorylates Chk2 on Thr68, leading to its activation and phosphorylation of several substrates, including p53, HDMX, PML (3), TRF2 (3,4) and KAP1 (5,6). Active Chk2 thus amplifies the DDR signal and promotes transient cell cycle arrest to allow DNA repair or, in presence of irreparable damages, the induction of apoptosis. The failure of these mechanisms leads to accumulation of genetic alterations, a common feature of cancer cells (7).

The 11S proteasome activator REGγ binds and activates the catalytic function of the 20S proteasome (8), promotes the ubiquitin-independent degradation of a number of proteins, including SRC-3, p21, p16 and p53 (8) and is involved in the regulation of chromosomal stability (9). Most recently, REGγ was found to be acetylated by CBP and deacetylated by SIRT1, respectively, promoting or inhibiting REGγ activity (10). Moreover, the interaction of REGγ with SIRT1 promotes its inhibition and Ub-independent degradation (11). In addition, we have demonstrated that REGγ physically binds Chk2 in human cells (12), whereas other studies have shown that REGγ is phosphorylated in an ATM-dependent manner following genotoxic stress (13).DBC1 (Deleted in Breast Cancer 1; KIAA1967; CCAR2) is a nuclear protein encoded by a gene originally described as homozigously deleted in some breast cancers (14–16). In spite of this deletion assignment, further studies showed DBC1 overexpression in breast, esophageal, gastric and colorectal cancers (17–21) and loss of DBC1 increases the death of breast cancer cells (22,23); however, DBC1 inhibits cancer cell survival following genotoxic stress (24,25). For these controversial effects on cancer cells, it is still unclear whether DBC1 acts as a tumor suppressor or a tumor promoter.

It is now well established that DBC1 interacts with and negatively regulates SIRT1 (24,25), a NAD^+^-dependent deacetylase able to deacetylate histone and non-histones protein, such as the tumor suppressor p53 (26,27). DBC1-mediated repression of SIRT1 results in increased levels of p53 acetylation and upregulation of p53-mediated apoptosis after DNA damage (24,25). Previously, we and others reported that, in response to DNA damage, DBC1 phosphorylation of Thr454 by ATM and ATR enhances DBC1 binding to and inhibition of SIRT1, promoting p53 activation and induction of apoptosis (28,29). Furthermore, recent studies show that, after DNA damage, kinase suppressor of Ras-I (KSR1) plays a role in decreasing DBC1 phosphorylation of Thr454, leading to a reduced DBC1-SIRT1 interaction and a lower transcriptional activity of p53 (30). Moreover, DBC1-SIRT1 binding is negatively regulated by hMOF acetylation (a member of the MYST family of histone acetyltransferases) of specific DBC1 residues, a process inhibited by ATM upon DNA damage (31). Collectively these results indicate that DBC1 and SIRT1 form, in human cells, a dynamic and regulated complex.

Here, we report that beside ATM and ATR, also Chk2 kinase and REGγ proteasome activator are required for SIRT1 inhibition by DBC1 and for the induction of DBC1-dependent apoptosis, in response to etoposide-induced DNA damage.

## MATERIAL AND METHODS

### Cell lines, treatments and antibodies

Human osteosarcoma U2OS cell line was cultured in DMEM (Dulbecco's modified Eagle's medium, Gibco), supplemented with 10% fetal bovine serum and 1% penicillin/streptomycin; cells were maintained at 37°C and 5% CO_2_. The proteasome inhibitor MG132 (Sigma) was added to cells 20 min before genotoxic treatments to a final 25 μM concentration. The Chk2 inhibitor VRX0466617 (kindly provided by Dr Minmin Yang, Pharmablock), was added 1 h before treatments at 100 μM. Etoposide (TEVA) was used at 20 μM and Neocarzinostatin (Sigma) at 8.8 nM. Cells viability was analyzed by trypan blue (Sigma) staining.

### Expression vectors, siRNAs and tranfections

Vectors encoding DBC1-WT and DBC1-T454A were reported in (28), FLAG-SIRT1, HA-DBC1-WT and deletion mutants plasmids were described in (24), whereas HA-Chk2 and FLAG-Chk2 were reported in (32). FLAG-REGγ was described in (12) and site directed mutagenesis to change the REGγ-S247 residue to A was performed with QuikChange II XL Site Directed Mutagenesis Kit (Stratagene) according to manufacturer protocol. siRNAs against DBC1, SIRT1, Chk2 and REGγ were ON-TARGETplus SMARTpool reagents and purchased from Thermo Scientific Dharmacon. Lipofectamine 2000 (Invitrogen) and Lipofectamine RNAiMAX (Invitrogen) were used for plasmids and siRNAs transfections, respectively.

### Western blots and immunoprecipitations

Western blot (WB) analyses were performed with the NuPAGE system (Invitrogen) and densitometric evaluations were done with the ImageQuant 5.2 software (Molecular Dynamics). REGγ phosphorylation analyses were performed using Phos-Tag (Wako) at 30 μM in a 10% acrylamide gel (13). The antibodies used were: DBC1 (Bethyl Laboratories, A300–434A or Cell Signaling Technology, clone 3G4); p53 (Santa Cruz, DO-7); p53-Ac-K382, phospho-Chk2-T68, PUMA, Cleaved PARP-1 and Cleaved Caspase-3 (Cell Signaling Technology); FLAG (clone M2), β-Actin and SIRT1 (Sigma); HA (clone 12CA5, Roche); REGγ (Transduction Laboratories). Chk2 antibody (clone 44D4/21) was previously described (33) or purchased from MBL Intl Corp (DCS-270 and DCS-273). Immunoprecipitation (IP) experiments were carried out as described (12) using cell lysates prepared in ELB buffer (150 mM NaCl, 50 mM Hepes pH 7.5, 5 mM EDTA, 0.5% NP40). After preclearing with protein-G (for mouse antibodies) or protein-A (for rabbit antibodies) coupled sepharose beads (Sigma), proteins of interest were subjected to IP with specific antibodies and analyzed by WB.

### Recombinant protein production and *in vitro* kinase assays

Recombinant GST-DBC1, GST-REGγ, His-SIRT1 proteins and fragments were produced in *Escherichia coli* and purified. *In vitro* kinase reactions with catalytically active GST-Chk2 were performed as previously reported (32).

### Pull down

REGγ-S247 non-phospho-peptides (EKIKRPR**S**SNAET LY) and phospho-peptides (EKIKRPR(p)**S**SNAETLY) conjugated to agarose beads were purchased from PrimmBiotech. Beads were incubated with cell lysates prepared in ELB buffer and, after washing, samples were boiled in SDS sample buffer and analyzed by western blot. For phosphopeptides dephosphorylation, FastAP Thermosensitive Alkaline Phosphatase (Fermentas) was used, according to manufacturer's protocol.

## RESULTS

### Chk2 kinase is required for DBC1-dependent SIRT1 inhibition

**Figure 1.**
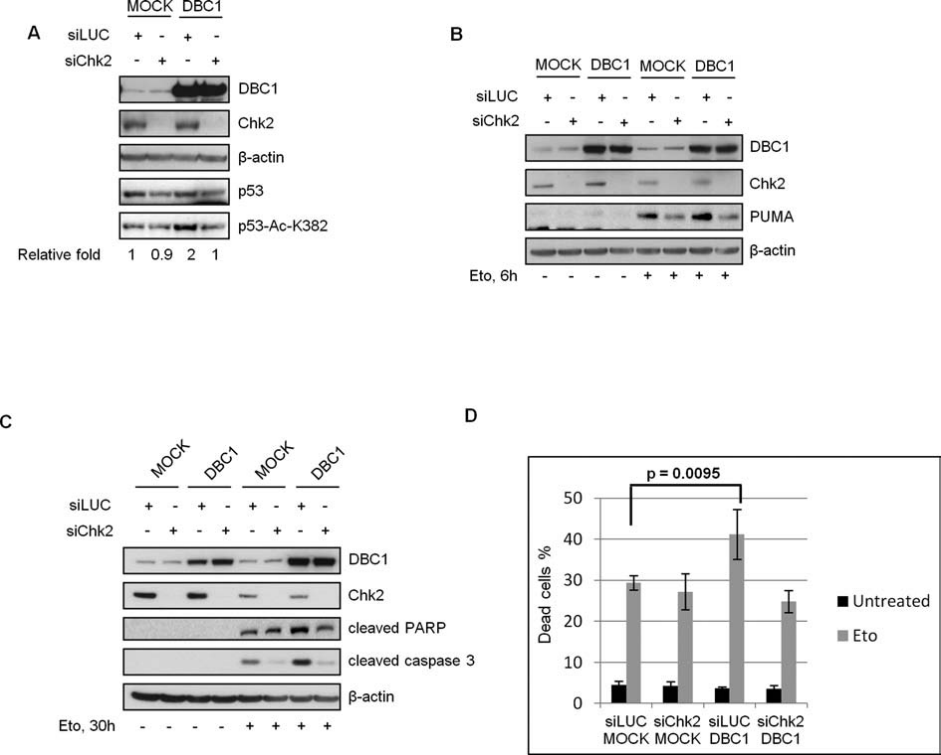
Chk2 is required for DBC1-dependent SIRT1 inhibition and induction of p53-mediated apoptosis after DNA damage in U2OS cells. Western blot analysis of total cell extracts from cells transfected with control or Chk2 siRNA and then with MOCK- or DBC1-encoding vectors. In (**A**) cells were incubated with 20 μM MG132 for 20 min prior to treatment with etoposide for 1 h and analyzed for p53 acetylation. Densitometric analyses (relative fold) show the ratios of acetylated p53-K382/total p53 normalized to the value of MOCK-transfected cells. In (**B**) and (**C**) cells were respectively treated with etoposide for 6 and 30 h and PUMA levels and apoptotic markers were analyzed. The same cells of experiment C were used to evaluate the percentage of dead cells by trypan blue staining. Values are mean ± SD from three independent experiments. Significant *P*-value is indicated (**D**).

We and others have previously shown that genotoxic stress induces the phosphorylation by ATM/ATR of DBC1-Thr454, promoting DBC1's interaction with and inhibition of SIRT1 deacetylase (28,29). As the checkpoint kinase Chk2, a target of ATM, catalyzes the phosphorylation of several proteins that are also phosphorylated by ATM (3), and upon oxidative stress promotes SIRT1 mRNA decay by phosphorylating the mRNA binding protein HuR (34), we asked whether Chk2 might also be involved in DBC1-dependent regulation of SIRT1. For this, the acetylation of p53, which we found to increase following DBC1 overexpression as result of SIRT1 inhibition (28), was analyzed in U2OS cells co-transfected with siRNA against Chk2 (or siLUC control sequence) and with a vector encoding DBC1 (or MOCK vector). Although in unstressed cells neither DBC1 overexpression nor Chk2 depletion affected p53 acetylation (data not shown), in etoposide-treated cells DBC1 overexpression raised p53 acetylation in Chk2-proficient, but not in Chk2-depleted cells (Figure 1A, compare lane 3 and 4). To verify that this event is not caused by Chk2-dependent induction of SIRT1 protein, possibly mediated by HuR, we performed western blot analysis of control and Chk2-depleted cells expressing ectopic DBC1, but no significant differences in SIRT1 protein levels were found among samples (Supplementary Figure S1).

As DBC1-induced p53 acetylation promotes p53-dependent transcriptional activity and apoptosis (28), we examined the effect of Chk2 depletion on these events. We found in etoposide-treated DBC1-overexpressing cells a marked PUMA accumulation and cleavage of both PARP-1 and Caspase-3 in Chk2-proficient cells, but not in Chk2-deficient cells (respectively, Figure 1B and C, compare lanes 5 and 7 and lanes 7 and 8). Concordant with these biochemical findings, the increased etoposide-induced cell death, due to DBC1 overexpression, was antagonized by Chk2 ablation (Figure 1D). Curiously, we also found that Chk2 silencing prevents PUMA induction and Caspase-3 cleavage (Figure 1B and C) and that Chk2 depletion alone cannot affect etoposide-induced apoptosis (Figure 1D).

Since also ectopic Chk2 can promote apoptosis (35,36), we verified whether the DBC1-induced cell death might be attributed to an increased Chk2 activity. However, we did not find any effect of DBC1 ablation on the rate of Chk2-induced cell death (Supplementary Figure S2). Notably, we found that DBC1 ablation did not reduce apoptosis induction, contrary to previous reports (24,25), even though p53 acetylation and PUMA expression were reduced (Supplementary Figure S2), and PARP cleavage induced (Supplementary Figure S2). The balance among these apoptotic factors could possibly explain why DBC1 absence does not affect cell death in our experimental conditions.

To determine whether Chk2 inhibition has the same effect on DBC1-SIRT1 regulation as Chk2 knockdown, experiments were performed in U2OS cells overexpressing DBC1 that, prior to etoposide exposure, were pre-treated with VRX0466617 (hereafter referred to VRX), a selective inhibitor of Chk2 (37). The results showed that the catalytic activity of Chk2 is indispensable for DBC1-mediated induction of p53 acetylation and apoptosis (Supplementary Figure S3).

Collectively, besides ATM/ATR, the activity of Chk2 kinase is also required for DBC1-mediated SIRT1 inhibition, ensuing acetylation/activation of p53, and finally apoptosis.

### Chk2 regulates DBC1-SIRT1 association

**Figure 2.**
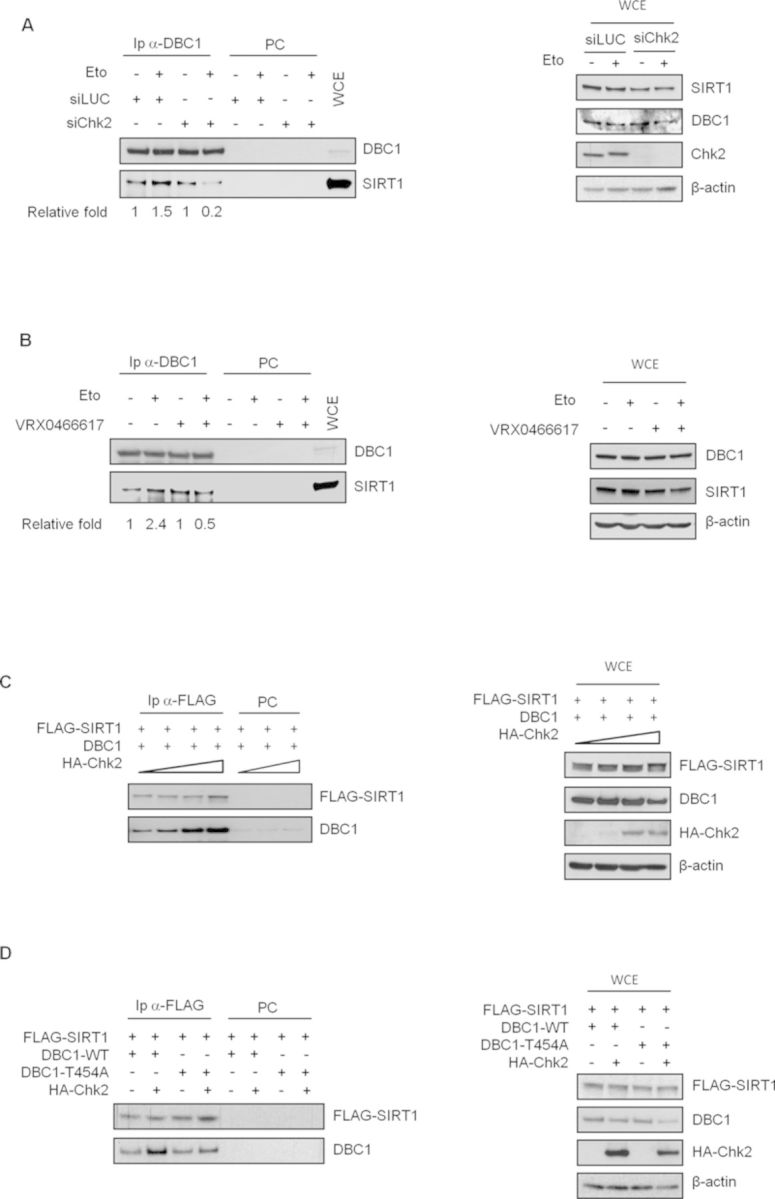
Chk2 kinase activity promotes DBC1-SIRT1 binding in response to DNA damage in U2OS cells. DBC1 was immunoprecipitated from cells transfected with control or Chk2 siRNA and treated or not with etoposide (**A**) or from cells pretreated with VRX and then exposed to etoposide (**B**); SIRT1 presence in immunocomplexes was determined by western blot (left) and total lysates (right) were analyzed for protein levels. Relative fold indicates the densitometric quantification of SIRT1 co-immunoprecipitated with DBC1; data from etoposide-treated cells were normalized to those from untreated samples. (**C**) Cells were transfected with DBC1, FLAG-SIRT1 and increasing levels of HA-Chk2-encoding vectors and FLAG-SIRT1 was immunoprecipitated; immunocomplexes (left) and protein levels in total cell extracts (left) were analyzed by western blot with the indicated antibodies. (**D**) Cells were transfected with DBC1-WT or T454A mutant, FLAG–SIRT1- and HA-Chk2-encoding vectors and FLAG-SIRT1 was immunoprecipitated; immunocomplexes (left) and protein levels in total cell extracts (right) were analyzed by western blot with the indicated antibodies. WCE, total cell extracts; IP, immunoprecipitates; PC, pre-cleared negative control.

To investigate the mechanism involved in SIRT1 inhibition by DBC1 and Chk2, we initially determined whether Chk2 depletion could affect DBC1 phosphorylation on Thr454, but, as shown in Supplementary Figure S4, no significant differences were found between control and siChk2 transfected cells before and after etoposide exposure. We then analyzed DBC1-SIRT1 association in response to DNA damage in control and Chk2 depleted U2OS cells. Although DBC1 and SIRT1 protein levels were similar in all cell extracts (Figure 2, right panel), we found that etoposide treatment increased the amount of SIRT1 that co-immunoprecipitated with DBC1, as already reported (28,29), but no increase was seen in Chk2-silenced cells (Figure 2A left, compare lane 2 with lane 4). Similar results were obtained analyzing the co-immunoprecipitation between DBC1 and SIRT1 in FLAG-DBC1 overexpressing cells depleted of Chk2 by siRNA (Supplementary Figure S5), thus excluding an influence of DBC1 expression levels on Chk2 regulation of DBC1-SIRT1 complex formation. In addition co-immunoprecipitation (co-IP) assays performed in the presence of VRX indicated that the catalytic activity of Chk2 is necessary for increasing DBC1-SIRT1 binding in response to etoposide (Figure 2B, left), even if VRX treatment alone seems to increase the association between these two proteins. However, since the DBC1-SIRT1 association is regulated also by phosphorylative events, it is possible that this phenomenon could be an effect of Chk2 inhibition on phosphatases activity as previously reported (38). To further verify the role of Chk2 in promoting DBC1-SIRT1 association, U2OS cells were transfected with fixed amounts of vectors encoding DBC1 and FLAG-SIRT1, and increasing amounts of a vector encoding HA-Chk2, which is known to homodimerize and become active when overexpressed (39). FLAG-SIRT1 immunocomplexes were then analyzed by western blot and, as expected, DBC1 binding to SIRT1 increased with the expression of Chk2 (Figure 2C, left). Similar experiments were performed to determine the role of Thr454 phosphorylation in Chk2-mediated increase of DBC1-SIRT1 association and we found that Chk2 overexpression cannot induce the binding of DBC1-T454A to SIRT1 (Figure 2D). Altogether, these findings suggest that Chk2 kinase promotes the physical association between DBC1 and SIRT1 in DNA damaged cells and, for this, the phosphorylation of DBC1 on Thr454 by ATM/ATR is also required.

**Figure 3.**
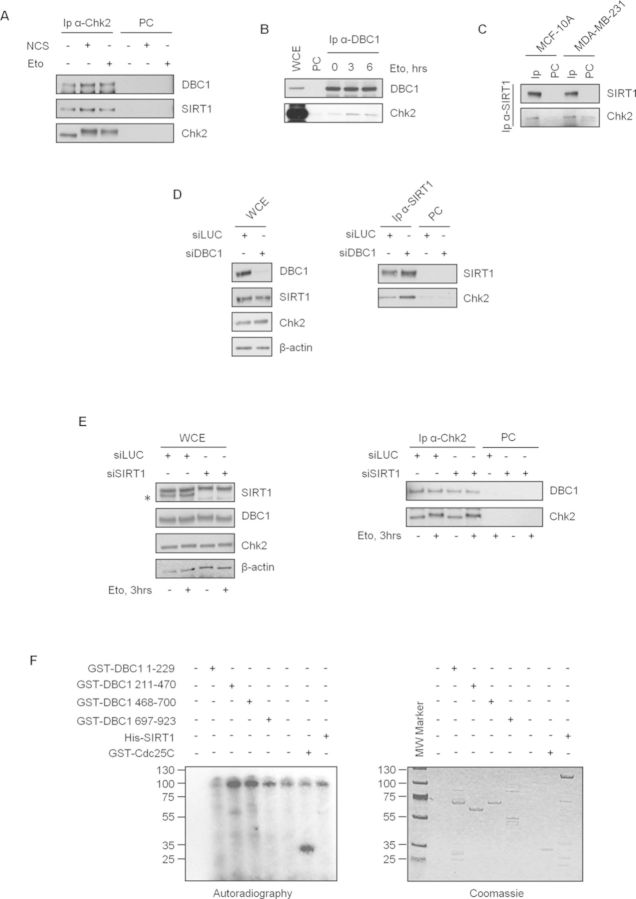
Chk2 interacts with DBC1 and SIRT1 in human cells. (**A**–**C**) Co-Ip experiments demonstrating the association of Chk2 with DBC1 and SIRT1, before and after DNA damage, in different cell lines. WCE, total cell extracts; IP, immunoprecipitates; PC, pre-cleared negative control. (**D**) U2OS cells were transfected with control or DBC1 siRNA and endogenous SIRT1 was immunoprecipitated. Total protein levels (left) and Chk2 protein in immunocomplexes (right) were determined by western blot. (**E**) Chk2 was immunoprecipitated from control or siSIRT1 transfected cells before and after etoposide exposure. Total protein levels and co-Ip with DBC1 were analyzed by WB. Asterisk indicates SIRT1 specific band. (F) *In vitro* kinase assays with recombinant active Chk2 and GST-DBC1 deletion mutants, His SIRT1 and GST-Cdc25C (positive control) as substrates.

**Figure 4.**
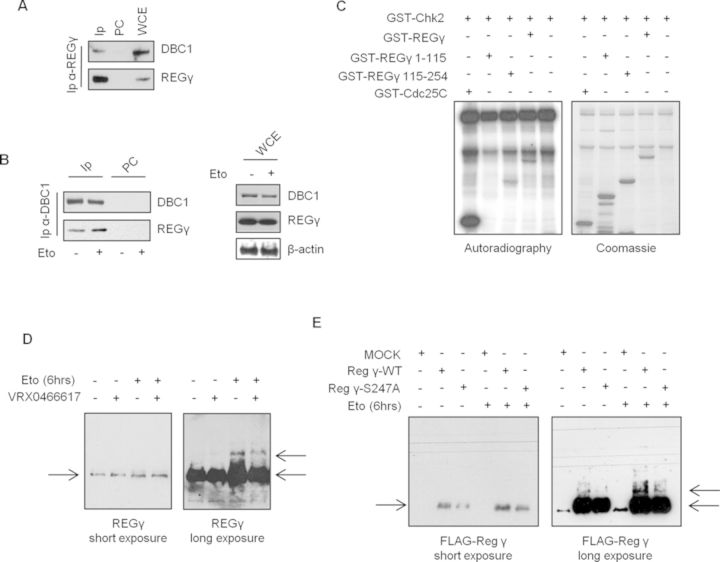
REGγ interacts with DBC1 and is phosphorylated by Chk2. (**A**) DBC1 is present in REGγ immunocomplexes from U2OS cells. (**B**) DBC1 was immunoprecipitated from U2OS cells before and after etoposide exposure. The presence of REGγ was determined by immunoblot. WCE, total cell extracts; IP, immunoprecipitates; PC, pre-cleared negative control. (**C**) *In vitro* kinase assays with recombinant active GST-Chk2 and full length or deletion mutants GST-REGγ as substrates. GST-Cdc25C was used as positive control. (**D**) Phos-Tag gel analyses of REGγ molecular shifts in response to etoposide and in the absence or presence of the Chk2 inhibitor VRX. (E) U2OS cells were transfected with vectors encoding FLAG-REGγ^WT^ and FLAG-REGγ^S247A^ and treated or untreated with etoposide. Molecular shifts of the ectopic proteins were analyzed by phos-tag gel.

### Chk2 binds DBC1 and SIRT1 in human cells

We next carried out co-IP experiments to determine whether Chk2 physically interacts with DBC1 and SIRT1 in human cells and to what extent DNA damage regulates this interaction. For this, Chk2 was immunoprecipitated from U2OS cells treated or untreated with neocarzinostatin or etoposide and immunoblotted for DBC1 and SIRT1, and, as shown in Figure 3A, Chk2 immunoprecipitates, but not negative controls, showed an interaction with both DBC1 and SIRT1 that weakly increased after DNA damage. Additional co-IP experiments confirmed the presence of Chk2 in DBC1 immunocomplexes from U2OS cells (Figure 3B), and in SIRT1 immunoprecipitates prepared from other two cell lines (Figure 3C).

We next analyzed whether DBC1 could affect Chk2-SIRT1 association. U2OS cells were therefore transfected with siRNA to knockdown DBC1 and SIRT1 immunocomplexes analyzed for Chk2. As shown in Figure 3D right, DBC1 depletion had no effect on Chk2-SIRT1 binding. Similarly, SIRT1 knockdown had no effect on DBC1-Chk2 association, either before or after DNA damage (Figure 3E).

Together, these co-IP studies are consistent with a model where DBC1, SIRT1 and Chk2 form a protein complex in human cells, and loss of any one of these proteins does not impair the binding between the other two.

We then asked whether DBC1 and SIRT1 could be candidate substrates for Chk2 kinase. To verify this, we initially set up *in vitro* kinase assays with recombinant GST-Chk2 (40) and GST-DBC1 as substrate, and although GST-Chk2 was able to autophosphorylate and to target a GST-Cdc25C fragment, used as positive control, GST-DBC1 not only remained unphosphorylated, but quite surprisingly inhibited Chk2 activities (Supplementary Figure S6). Similar results were obtained using His-DBC1 (data not shown), indicating that this inhibitory effect on Chk2 kinase is not due to GST-induced multimerization. Other *in vitro* Chk2 kinase assays performed with four DBC1 deletion fragments and full length SIRT1 showed no significant phosphorylation of these recombinant substrates (Figure 3F), but they allowed to map the N-terminus sequence (a.a. 1–229) of DBC1 as being responsible for the inhibition of Chk2 autophosphorylation and activity towards Cdc25C (Figure 3F, lane 1). It should be noted however that, in cells, deletion of the N-terminus region of DBC1 (a.a. 1–179) is sufficient to abolish DBC1 binding to Chk2 which maps on Chk2 catalytic domain (Supplementary Figure S7).

Collectively, these data indicate that, at least *in vitro*, DBC1 and SIRT1 are not substrates of Chk2 kinase and that the N-terminus of DBC1 inhibits the activity of Chk2 by binding to its kinase domain.

### REGγ proteasome activator interacts with DBC1 and is a Chk2 phosphorylation target

We previously demonstrated that Chk2 interacts with the proteasome activator REGγ (12), while, more recently, it was shown that SIRT1 deacetylates REGγ and reduces its activity (10), and that the interaction of these proteins promotes SIRT1 degradation and inhibition by substrates displacement (11). These observations raise the possibility of interplay between REGγ, Chk2, SIRT1 and DBC1. To test this, we initially verified whether DBC1 and REGγ associate in human cells and found, in co-IP assays, that endogenous REGγ and DBC1 reciprocally immunoprecipitate each other (Figure 4A and B) both before and after DNA damage (Figure 4B).

As REGγ protein contains two predicted consensus motifs for Chk2 phosphorylation on Ser24 and Ser247 (Supplementary Figure S8), we checked in *in vitro* kinase assays the activity of Chk2 towards full length and deleted forms of recombinant GST-REGγ. The results (Figure 4C) evidenced a phosphorylation signal on GST-REGγ and GST-REGγ_115–254_ fragment, but not on GST-REGγ_1–115_, suggesting Ser247 as a Chk2 phosphosite. Phos-tag gel analysis previously demonstrated that genotoxic damage induces an ATM-dependent phosphorylation of REGγ (13). To determine whether Chk2 also phosphorylates REGγ *in vivo*, cell extracts prepared from etoposide-treated or untreated samples, with or without the Chk2 inhibitor VRX, were examined with the Phos-tag western blot technique. The results show in etoposide-treated cells the presence of a slower migrating REGγ form (Figure 4D), in accordance with earlier findings (13), markedly attenuated by VRX. The absence of this form in cells where REGγ was knocked down by siRNA validated the results (Supplementary Figure S9). We also analyzed the mobility shift of FLAG-REGγ^WT^ and FLAG-REGγ^S247A^ and found that the intensity of the slower migrating band is strongly attenuated in cells expressing FLAG-REGγ^S247A^ mutant protein (Figure 4E). Together, these results indicate that DBC1 and REGγ interact in human cells and that Chk2 can phosphorylate a fraction of the total pool of REGγ in human cells on Ser 247.

### SIRT1 inhibition by DBC1 is REGγ-dependent

**Figure 5.**
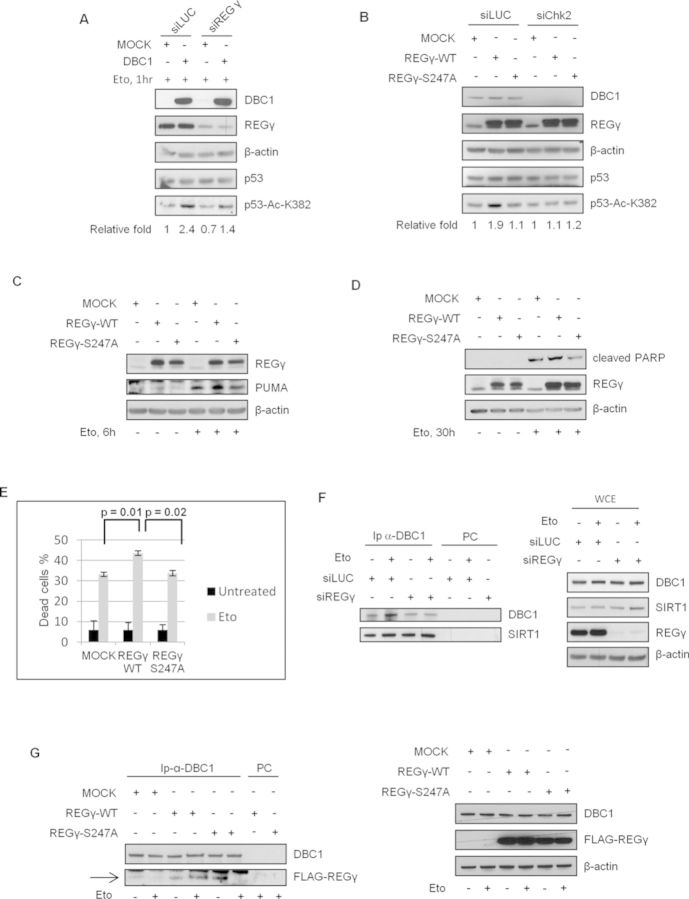
REGγ mediates DBC1 and Chk2-dependent SIRT1 inhibition. (**A**) U2OS cells were transfected with control or REGγ siRNA, then with MOCK- or DBC1-encoding vectors and exposed to etoposide for 3 h. p53-acetylation levels were determined by western blot. (**B**) Control or Chk2 silenced cells overexpressing FLAG-REGγ^WT^ or FLAG-REGγ^S247A^ were expose to etoposide and analyzed for p53 acetylation. In (A) and (B) relative fold indicates the ratios of acetylated p53-K382/total p53 normalized to the value of MOCK-transfected cells. In (**C**) and (**D**) cells were transfected with MOCK-, FLAG-REGγ^WT^- and FLAG-REGγ^S247A^-encoding vectors treated respectively with etoposide for 6 and 30 h and PUMA levels and apoptotic markers were analyzed. (**E**) The same cells of experiment (D) were used to evaluate the percentage of dead cells by trypan blue staining. (**F**) Co-IP experiments of DBC1 and SIRT1 in REGγ-depleted cells (left) and western blot analysis of total protein levels in the same extracts (right) (**G**) Co-IP experiments of DBC1 and FLAG-REGγ^WT^ or FLAG-REGγ^S247A^ (left) and western blot analysis of total protein levels in the same extracts (right). Arrow indicates FLAG-positive bands.

The potential role played by REGγ in DBC1-dependent inhibition of SIRT1 was analyzed in DBC1-overexpressing U2OS cells transfected with siRNA against REGγ or a control sequence, and the results showed that REGγ depletion reduces the DBC1-dependent increase in p53 acetylation (Figure 5A). Moreover, when we overexpressed REGγ^WT^ or REGγ^S247A^ in control or Chk2 silenced U2OS cells, we detected an increase in p53 acetylation, upon etoposide, only with the ectopic wild-type protein and in Chk2 proficient cells (Figure 5B), clearly indicating that SIRT1 inhibition by DBC1 requires REGγ phosphorylated on S247 by Chk2. Accordingly, when we analyzed the role of REGγ phosphorylation in PUMA protein induction and apoptosis, we found that the expression of REGγ^WT^, but not of REGγ^S247A^, can promote the accumulation of PUMA and the increase of U2OS cell death (Figure 5C–E). To further investigate the mechanism underlying REGγ mediated DBC1-dependent SIRT1 inhibition, we analyzed DBC1-SIRT1 complex in REGγ silenced cells. We found that, like Chk2 depletion, REGγ silencing prevents the increase of the association between DBC1 and SIRT1 upon etoposide exposure (Figure 5F).

We then determined the function of REGγ phosphorylation on Ser247 in DBC1-REGγ complex formation and found that, whereas the binding of DBC1 to REGγ^WT^ increases in response to DNA damage, the association with REGγ^S247A^ is reduced (Figure 5G left, compare lanes 4 and 6). To further address this point we performed pull-down assays by incubating cell lysates from untreated and etoposide treated cells with REGγ peptides phosphorylated or not on Ser247 (respectively, REGγ-pS247 and REGγ-S247). We found that DBC1 binds better the non-phosphorylated peptide than the phosphorylated one, but whereas the association between DBC1 and REGγ-S247 is reduced upon DNA damage, the binding of DBC1 with REGγ-pS247 is maintained in response to etoposide treatment (Supplementary Figure S10). Similar results were obtained also analyzing the binding of DBC1 to the REGγ phosphorylated peptide that had been pretreated with phosphatase (Supplementary Figure S10). Collectively these data suggest that REGγ-Ser247 phosphorylation promotes its association with DBC1 and contributes to the DBC1-mediated inhibition of SIRT1.

## DISCUSSION

Here, we report that catalytically active Chk2 is required for DBC1-mediated SIRT1 deacetylase inhibition and p53-dependent apoptosis. This phenomenon is not achieved through Chk2-dependent alterations of DBC1 phosphorylation on Thr454, but through a mechanism in which Chk2 potentiates the DBC1-SIRT1 binding and consequently SIRT1 inhibition. Indeed this event is abolished by depletion or chemical inhibition of Chk2 and, as we demonstrated, it is not influenced by DBC1 expression levels, even if we found that Thr454 phosphorylation is necessary for the Chk2 mediated increase of DBC1-SIRT1 binding. However, although we noticed in Chk2-depleted cells reduced level of both PUMA protein and cleaved caspase-3, as previously reported in (41,42), we curiously found that Chk2 depletion or chemical inhibition has no effect on etoposide-induced p53 acetylation and apoptosis, as demonstrated by cleaved PARP1 levels and by the percentage of dead cells. This event could possibly be explained by the redundancy of cell cycle checkpoint pathways that could mask the apoptotic defect caused by Chk2 inhibition, as previously reported in normal cells for VRX treatment (37). Alternatively, we can also suggest the existence of a Chk2 independent way to etoposide-induced apoptosis, which does not require caspase-3 cleavage or the induction of PUMA protein. Instead, the increased cell death detectable upon DBC1 overexpression and etoposide exposure is dependent on Chk2 and established on a pathway which requires PUMA induction and caspase-3 cleavage. This route seems to be specifically based on the increase of p53 acetylation by DBC1-dependent SIRT1 inhibition. It should be noted, however, that neither DBC1 nor SIRT1 appeared to be phosphorylated by Chk2 *in vitro*, although we cannot definitely exclude the occurrence of these events *in vivo*, where other factors might favor Chk2 activity toward these substrates. Curiously, when we analyzed the role of DBC1 in Chk2 induced apoptosis, we found that DBC1 depletion does not affect cell death, as previously reported, although it reduces p53 acetylation and PUMA induction, but not PARP1 cleavage. This phenomenon might be ascribed to different experimental conditions, which can result in different effects of DBC1 depletion. Indeed, recently, other papers reported that DBC1 absence, in response to various stimuli, enhances cell death (43–45), thus supporting a model in which DBC1 can modulate apoptosis depending on the cellular context, type of damage and on the duration of genotoxic stress.

It was recently found that the activity of the 11S proteasome activator REGγ is regulated by CBP and SIRT1-dependent acetylation/deacetylation (10) and that REGγ itself is implicated in SIRT1 degradation and inhibition (11). Since we have previously found that REGγ binds Chk2 (12), we postulated that this protein could be the target of Chk2 activity involved in SIRT1 inhibition by DBC1. In accordance with this, we found that, upon DNA damage Chk2 phosphorylates REGγ on Ser247, both *in vitro* and *in vivo*, and that this modification promotes p53 acetylation, PUMA protein induction and apoptosis. Indeed, although REGγ was found not to be implicated in the regulation of SIRT1 activity towards p53 (11), in our cellular system we demonstrated that REGγ and its phosphorylation on Ser247 by Chk2 are necessary for the DBC1-dependent increase of p53-acetylation, further corroborating the hypothesis that this proteasome activator subunit could be the missing link between DBC1 and Chk2 toward SIRT1 activity inhibition. Additionally, we found that REGγ, like Chk2 and DBC1-Thr454 phosphorylation, is required to increase DBC1-SIRT1 binding and SIRT1 activity inhibition and that REGγ phosphorylation by Chk2 is necessary to increase REGγ-DBC1 binding in response to DNA damage. Accordingly, pull down experiments with phosphorylated and unphosphorylated peptides spanning REGγ-Ser247 indicated that the association of DBC1 with the non-phosphorylated peptide decreases upon etoposide treatment, whereas the binding with the peptide phosphorylated on Ser247, although strongly reduced, is not affected by DNA damage treatment. These results suggest that REGγ-Ser247 phosphorylation by Chk2 can induce a conformational change in REGγ protein that favors its binding to DBC1. Alternatively it is also possible that this phosphorylation could promote REGγ interaction with other protein(s) that then lead to the increased DBC1-REGγ association. The strong interaction between DBC1 and REGγS247A detectable in untreated cells could be due to a better affinity of the unphosphorylated REGγ for the DBC1 form present in untreated cells, as revealed also by the pull down assay. Indeed it is possible that overexpressed REGγ-WT could be partially phosphorylated on Ser247, like other overexpressed phosphoproteins, and that this phosphorylation could affect its association with DBC1 in untreated cells. Upon DNA damage, both DBC1 and REGγ are characterized by post-translational modifications and the modified DBC1 could strongly interact with the phosphorylated REGγ, but not with the unphosphorylated protein.

**Figure 6.**
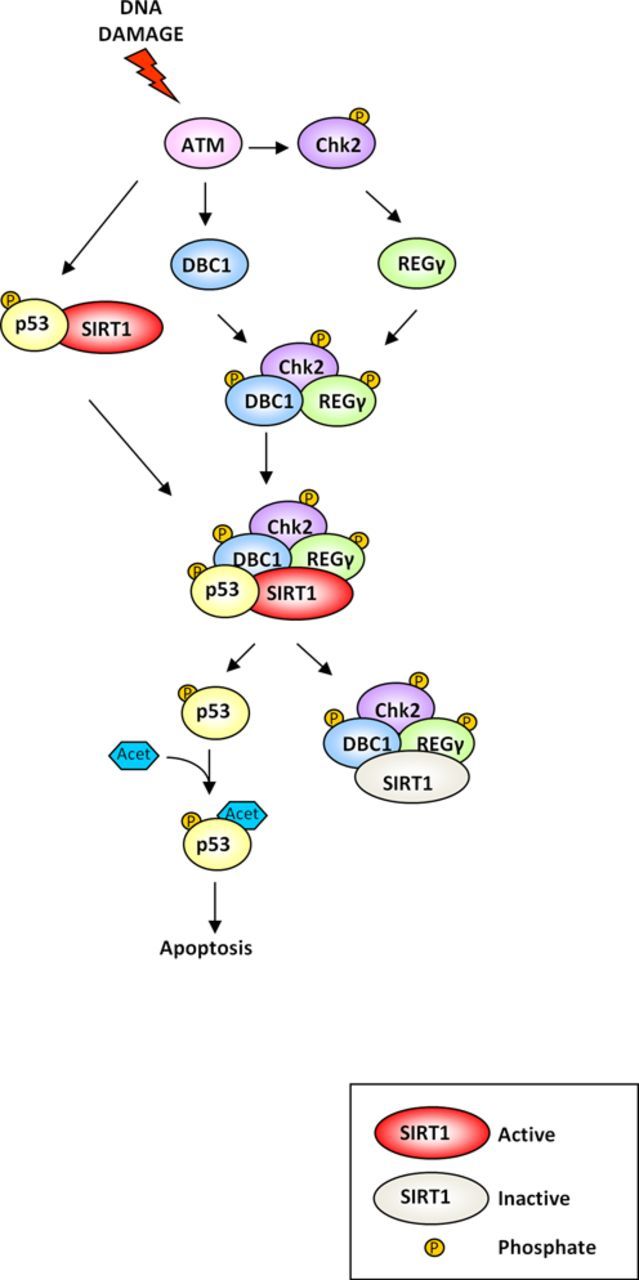
Graphical representation of DBC1 regulation in the DDR. In response to DNA damage ATM contributes to p53 activation in different ways, by phosphorylation of (i) p53 on Ser15, (ii) DBC1 on Thr454 and (iii) Chk2 on Thr68. Chk2, activated by ATM, then phosphorylates REGγ, promoting DBC1-REGγ association and SIRT1 inhibition. These events finally lead to p53 activation and p53-dependent apoptosis.

Thus, according to our model, following DNA damage ATM phosphorylates p53-S15, DBC1-T454 and Chk2-T68, inducing p53 stabilization, DBC1-SIRT1 binding and Chk2 activation and phosphorylation of REGγ-Ser247. These events enhance DBC1-REGγ complex formation which, in turn, further increases its binding to and inhibition of SIRT1, finally leading to p53-dependent apoptosis (Figure 6).

In summary, we demonstrated that DBC1 promotes apoptosis by SIRT1 inhibition and that this phenomenon is regulated by the ATM-Chk2-REGγ axis (Figure 6). With these results we shed new light into the role of DBC1 in the cellular response to DNA damage.

## SUPPLEMENTARY DATA

Supplementary Data are available at NAR Online.

SUPPLEMENTARY DATA
