# Supplementary material for: Chk2 and REGγ-dependent DBC1 regulation in DNA damage induced apoptosis
Source: Nucleic Acids Res. 2014 Oct 31;42(21):13150–60. doi: 10.1093/nar/gku1065 (PMC4245943; doi:10.1093/nar/gku1065)
Supplement: SUPPLEMENTARY DATA [file supp_42_21_13150__index.html]

Chk2 and REGγ-dependent DBC1 regulation in DNA damage induced apoptosis — Chk2 and REGγ-dependent DBC1 regulation in DNA damage induced apoptosis — SUPPLEMENTARY DATA 

# Chk2 and REGγ-dependent DBC1 regulation in DNA damage induced apoptosis

## SUPPLEMENTARY DATA

**Files in this Data Supplement:**

- SUPPLEMENTARY DATA
