# Supplementary material for: Chk2 and REGγ-dependent DBC1 regulation in DNA damage induced apoptosis
Source: Nucleic Acids Res. 2014 Oct 31;42(21):13150–60. doi: 10.1093/nar/gku1065 (PMC4245943; doi:10.1093/nar/gku1065)

SUPPLEMENTARY FIGURES LEGENDS

Supplementary Figure 1. Chk2 silencing and DBC1 overexpression do not affect SIRT1 protein levels. Western blot analysis of SIRT1 protein in cells transfected with control or Chk2 siRNA and then with mock or DBC1 encoding vectors.

Supplementary Figure 2. Chk2-induced apoptosis is not mediated by DBC1. (A) U2OS cells were transfected with control or DBC1 siRNA, then with mock or Chk2 encoding vectors and exposed or not to etoposide for 30 h. The percentage of dead cells was determined by Trypan blue exclusion staining. Values are mean and standard deviations of three independent experiments. U2OS cells were transfected with control or DBC1 silencing and assayed by western blot for p53 acetylation (B), PUMA protein levels (C) and PARP1 cleavage (D) upon the indicated etoposide treatment.

Supplementary Figure 3. Chk2 activity is required for DBC1-dependent SIRT1 inhibition and induction of p53-mediated apoptosis. Western blot analysis of total cell extracts from cells transfected with MOCK or DBC1 encoding vectors and treated with etoposide in the presence or absence of the Chk2 inhibitor VRX. In A cells were incubated with VRX for 1 hr and MG132 for 20 minutes prior to etoposide exposure and analyzed for p53 acetylation. In B and C cells were respectively incubated with VRX for 1hr and then with etoposide for 6 and 30 hours and PUMA levels and apoptotic markers were analyzed. Phosphorylation of Chk2-T68 indicates VRX efficacy [37]. The same cells of experiment (C) were used to evaluate the percentage of dead cells by trypan blue staining. Values are mean ± SD from three independent experiments. Significant p-value is indicated (D).

Supplementary Figure 4. Chk2 depletion does not affect DBC1 phosphorylation on T454 following etoposide exposure. U2OS cells were transfected with control or Chk2 siRNA and treated with etoposide for 1 and 3 h. Phosphorylation of DBC1-T454 was analyzed by western blot.

Supplementary Figure 5. Analysis of SIRT1 and ectopic DBC1 interaction in control and Chk2 depleted cells. U2OS cells, transfected with control or Chk2 siRNA and with mock or FLAG-DBC1 encoding vectors, were treated with etoposide for 3 h and FLAG-DBC1 was immunoprecipitated. The presence of SIRT1 in immunocomplexes was analyzed by western blot (left); total cell extracts were analyzed for protein levels (right).

Supplementary Figure 6. DBC1 inhibits *in vitro* Chk2 activity. In vitro kinase assays with recombinant active GST-Chk2 and GST-DBC1 or GST-Cdc25C (positive control) as substrates. Coomassie staining (left) shows protein levels and autoradiography (right) the phosphorylated proteins.

Supplementary Figure 7. The N-terminus of DBC1 interacts with Chk2 kinase domain. (A) Wild type DBC1 and deletion mutants were expressed in U2OS cells and immunoprecipitated with anti-HA antibody. The presence of Chk2 in the immunocomplexes was determined by western blot. Lanes corresponding to an unrelated experiment have been cropped. (B) Wild type Chk2 and deletion mutants were expressed and immunoprecipitated. DBC1 presence in immunocomplexes (left) and protein levels in total cell extracts (right) were analyzed by western blot. Ip, immunoprecipitates; PC, preclearing negative control; WCE, total cell extracts; IgG immunoglobulins.

Supplementary Figure 8. REGγ protein has two consensus sites for Chk2 activity. Table showing the two putative Chk2 consensus sequences (S24 and S247) on REGγ protein as determined by Motif Scan analyses .

Supplementary Figure 9. Specificity of REGγ shifted band. Control or siREGγ tranfected cells were incubated or not with VRX prior to be treated or untreated with etoposide. Extracts were then analyzed for REGγ molecular shifts by phos-tag gel. Lanes corresponding to an unrelated experiment have been cut.

Supplementary Figure 10. DBC1 pull down with REGγ peptides. U2OS cells were treated or not with etoposide for 3 h and total cell extracts were incubated with the agarose beads conjugated to REGγ-S247 non-phosphopeptide (S247), REGγ-S247 phosphopeptide (pS247) and REGγ-S247 phosphopeptide previously dephosphorylated with the FastAP Thermosensitive Alkaline Phosphatase (pS247 deP). Proteins bound to the beads were analyzed by western blot for the presence of DBC1.

Supplementary Figure 1

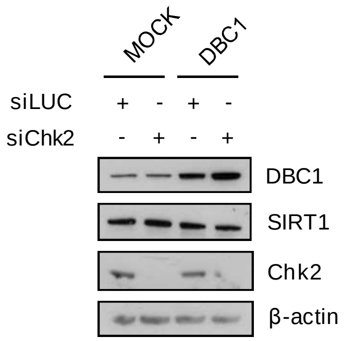

Supplementary Figure 2

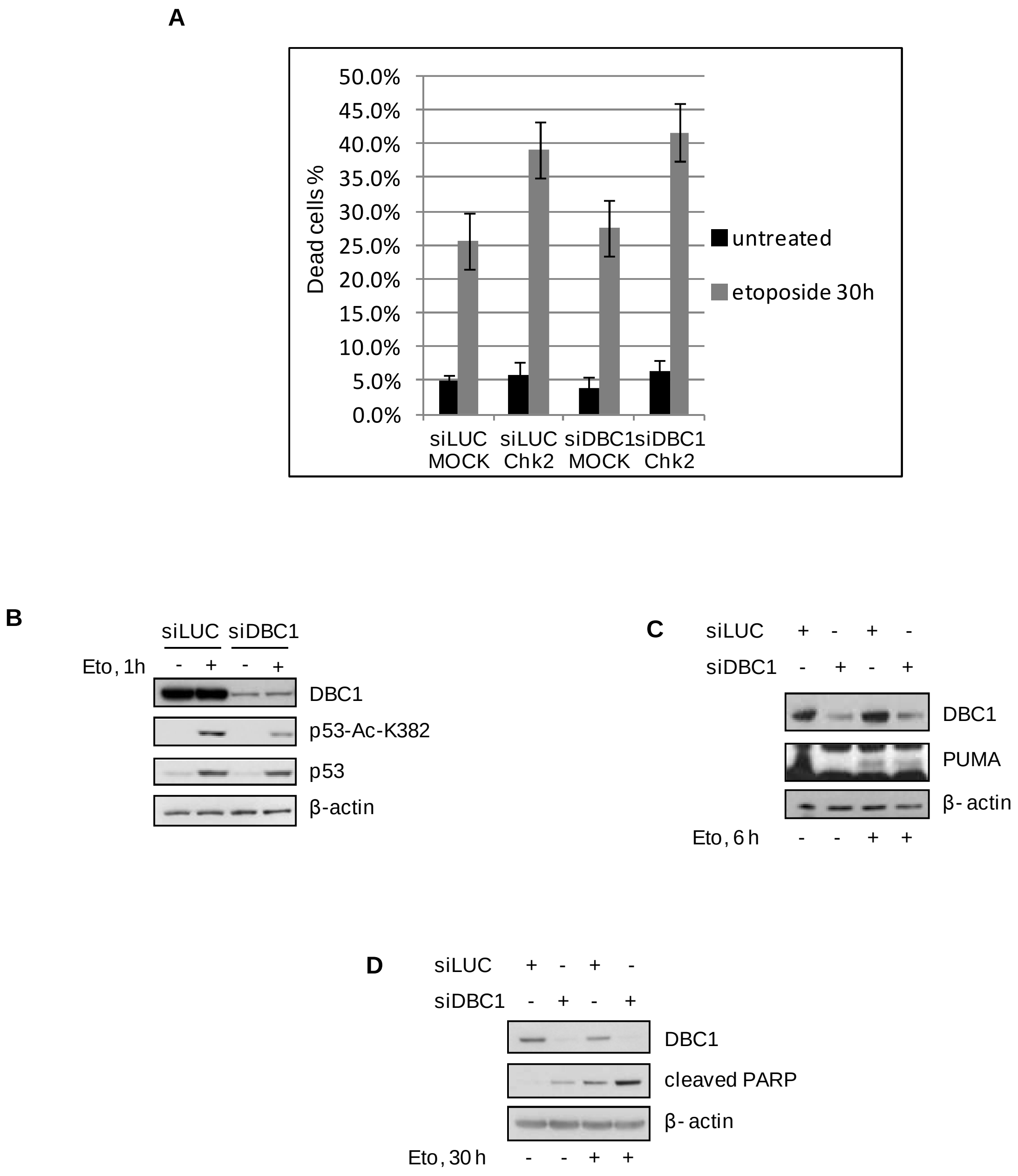

Supplementary Figure 3

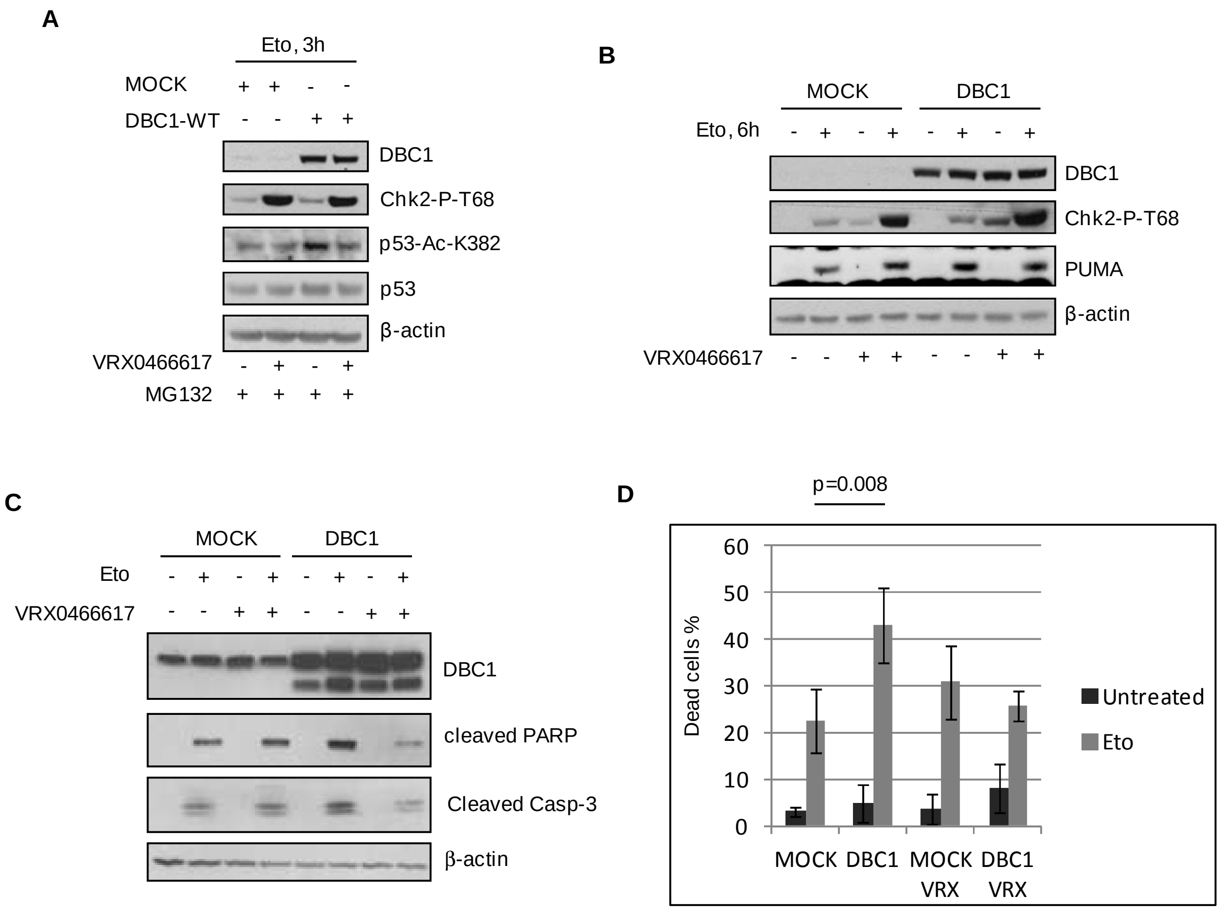

Supplementary Figure 4

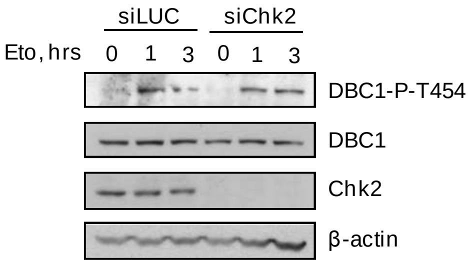

Supplementary Figure 5

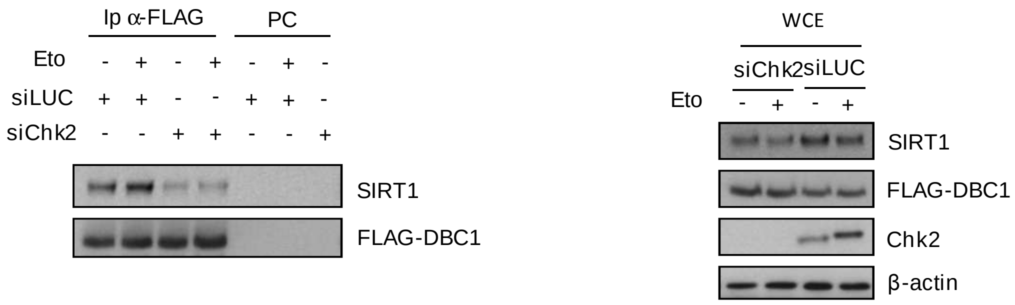

Supplementary Figure 6

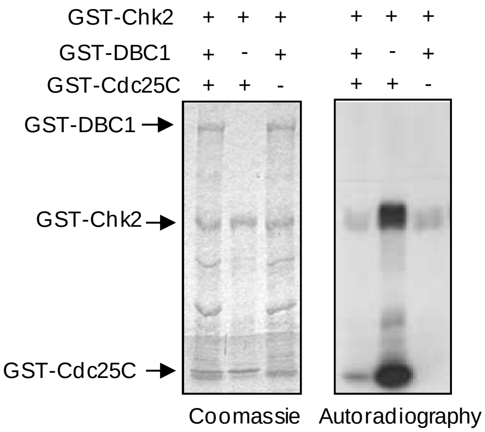

Supplementary Figure 7

A

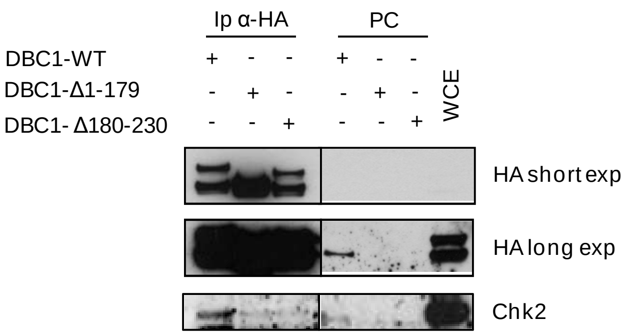

B

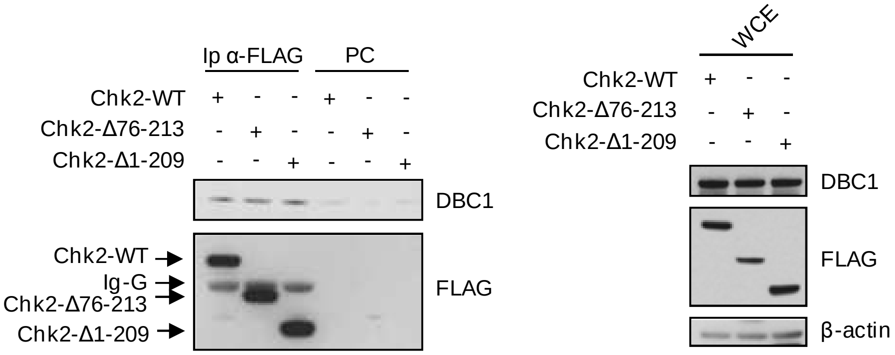

Supplementary Figure 8

Site	Score	Sequence
Ser24	1.0000	SFRERIT**S**EAEDLVA
Ser247	0.7692	EKIKRPR**S**SNAETLY

Supplementary Figure 9

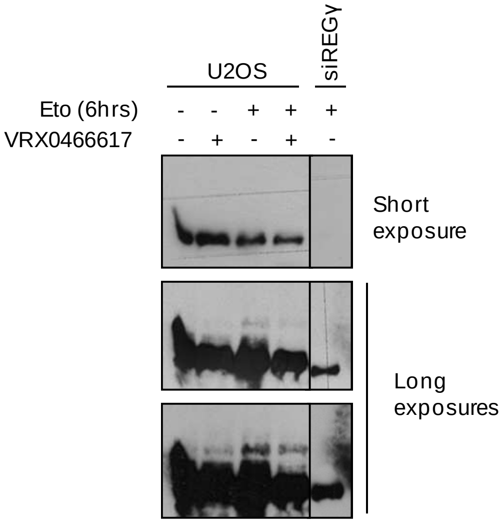

Supplementary Figure 10

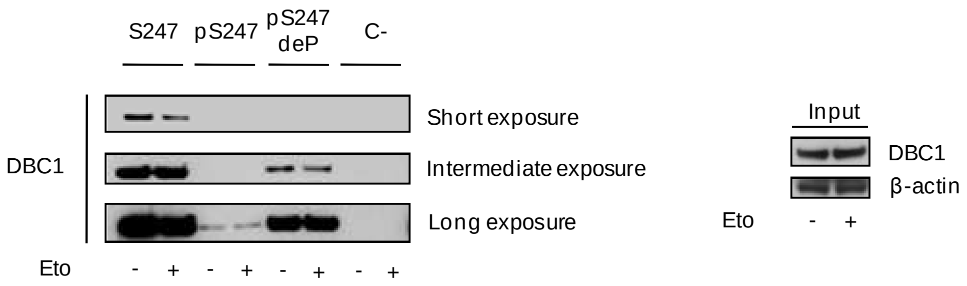

Supplement: SUPPLEMENTARY DATA [file supp_gku1065_nar-02893-d-2014-File008.pdf]
